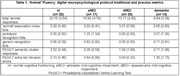# A Brief Digital Neuropsychological Protocol: III ‐ Using Artificial Intelligence to Measure Semantic Memory with the ‘Animal’ Fluency Test

**DOI:** 10.1002/alz.091512

**Published:** 2025-01-09

**Authors:** David J. Libon, Rodney Swenson, Sean Tobyne, Catherine C. Price, Melissa Lamar, Stephanie Cosentino, Russell Banks, Ali Jannati, John Showalter, David Bates, Alvaro Pascual‐Leone

**Affiliations:** ^1^ Rowan University, Stratford, NJ USA; ^2^ University of North Dakota School of Medicine and Health Sciences, Grand Forks, ND USA; ^3^ Linus Health, Boston, MA USA; ^4^ University of Florida, Gainesville, FL USA; ^5^ Department of Psychiatry and Behavioral Sciences, Rush University Medical Center, Chicago, IL USA; ^6^ Rush Alzheimer's Disease Center, Chicago, IL USA; ^7^ The Taub Institute for Research on Alzheimer’s Disease and the Aging Brain, Columbia University, New York, NY USA; ^8^ Columbia University Irving Medical Center, New York, NY USA; ^9^ Harvard Medical School, Boston, MA USA; ^10^ Department of Neurology, Harvard Medical School, Boston, MA USA; ^11^ Hinda and Arthur Marcus Institute for Aging Research, and Deanna and Sidney Wolk Center for Memory Health, Hebrew Senior Life, Boston, MA USA

## Abstract

**Background:**

Semantic memory refers to knowledge of attributes associated with common objects. Quantifying the strength of semantic association between successive ‘animal’ fluency responses can be challenging. The current research assessed between‐group differences for ‘animal’ fluency total output and selected verbal serial list learning, episodic memory measures.

**Method:**

Memory clinic patients were assessed with a digital neuropsychological protocol. Cluster analysis classified patients into normal (nl= 23), amnestic MCI (aMCI= 17), dysexecutive MCI (dMCI= 23), and dementia (dementia= 14) groups. During the protocol, patients were given 60secs to provide animal exemplars. Memory was assessed with the P(r)VLT, a 6‐word verbal serial list learning test. Using artificial intelligence assisted scoring all ‘animal’ fluency and P(r)VLT outcome variables including the ‘animal’ Association Index (AI), where the mean number of shared attributes between successive responses were automatically tallied.

**Result:**

The nl group generated more animal exemplars than all other groups (p< 0.001; Table 1). aMCI and dMCI patients generated more responses than dementia patients (p< 0.043, both analyses). NL and dMCI patients produced a higher, more semantically connected, ‘animal’ AI than dementia patients (nl > dem; p< 0.025, both analyses). Regression analysis (dv= ’animal’ AI; block 1= age, education, sex; block 2= recognition prototypic & generic foils) was significant (R^2^= 0.145, p< 0.023); and found that a reduced, more impaired ‘animal’ AI was associated with increasing numbers of P(r)VLT prototypic recognition foils (beta= ‐0.276; p< 0.024). Regression analysis (dv= ’animal’ AI; block 1= age, education, sex; block 2= P(r)VLT semantic cluster responses, P(r)VLT extra‐list intrusion errors) was not significant.

**Conclusion:**

In addition to commonly used outcome measures such as total ‘animal’ responses, this digital neuropsychological protocol scores a number of process variables, including the ‘animal’ AI. The association between reduced ‘animal’ AI and greater numbers of list learning prototypic recognition foils suggests combined episodic memory and semantic‐related impairment in selected patients. When brought to scale, automated analysis of neuropsychological process variables may aide in identifying emergent neurodegenerative illness.